# Comparison of the antibacterial activity of essential oils and extracts of medicinal and culinary herbs to investigate potential new treatments for irritable bowel syndrome

**DOI:** 10.1186/1472-6882-13-338

**Published:** 2013-11-28

**Authors:** Aiysha Thompson, Dilruba Meah, Nadia Ahmed, Rebecca Conniff-Jenkins, Emma Chileshe, Chris O Phillips, Tim C Claypole, Dan W Forman, Paula E Row

**Affiliations:** 1 Biochemistry Group, College of Medicine, Care of Grove Reception, Swansea University, Singleton Park, Swansea SA2 8PP, UK; 2 Welsh Centre for Printing and Coating, College of Engineering, The Talbot Building, Swansea University, Singleton Park, Swansea SA2 8PP, UK; 3 College of Science, The Wallace Building, Swansea University, Singleton Park, Swansea SA2 8PP, UK

**Keywords:** Irritable bowel syndrome, IBS, Small intestinal bacterial overgrowth, SIBO, Herbal medicine, Antibacterial, Antimicrobial, Essential oil

## Abstract

**Background:**

Irritable bowel syndrome (IBS) is a common functional gastrointestinal disorder, which may result from alteration of the gastrointestinal microbiota following gastrointestinal infection, or with intestinal dysbiosis or small intestinal bacterial overgrowth. This may be treated with antibiotics, but there is concern that widespread antibiotic use might lead to antibiotic resistance. Some herbal medicines have been shown to be beneficial, but their mechanism(s) of action remain incompletely understood. To try to understand whether antibacterial properties might be involved in the efficacy of these herbal medicines, and to investigate potential new treatments for IBS, we have conducted a preliminary study *in vitro* to compare the antibacterial activity of the essential oils of culinary and medicinal herbs against the bacterium, *Esherichia coli*.

**Methods:**

Essential oils were tested for their ability to inhibit *E. coli* growth in disc diffusion assays and in liquid culture, and to kill *E. coli* in a zone of clearance assay. Extracts of coriander, lemon balm and spearmint leaves were tested for their antibacterial activity in the disc diffusion assay. Disc diffusion and zone of clearance assays were analysed by two-tailed t tests whereas ANOVA was performed for the turbidometric assays.

**Results:**

Most of the oils exhibited antibacterial activity in all three assays, however peppermint, lemon balm and coriander seed oils were most potent, with peppermint and coriander seed oils being more potent than the antibiotic rifaximin in the disc diffusion assay. The compounds present in these oils were identified by gas chromatography mass spectrometry. Finally, extracts were made of spearmint, lemon balm and coriander leaves with various solvents and these were tested for their antibacterial activity against *E. coli* in the disc diffusion assay*.* In each case, extracts made with ethanol and methanol exhibited potent antibacterial activity.

**Conclusions:**

Many of the essential oils had antibacterial activity in the three assays, suggesting that they would be good candidates for testing in clinical trials. The observed antibacterial activity of ethanolic extracts of coriander, lemon balm and spearmint leaves suggests a mechanistic explanation for the efficacy of a mixture of coriander, lemon balm and mint extracts against IBS in a published clinical trial.

## Background

Irritable bowel syndrome (IBS) is a functional gastrointestinal disorder, which affects 10 – 22% of the UK population and is responsible for 20 – 50% of the workload of gastroenterology departments [1]. IBS is characterised by altered bowel habit, pain, and wind or bloating, which all severely affect quality of life and may last for decades. IBS can be classified into subgroups depending on the predominant bowel symptom, namely constipation-predominant (IBS-C), diarrhoea-predominant (IBS-D) or alternating between the two (IBS-A) [2,3]. Post-infectious (PI) IBS may arise after an episode of acute gastroenteritis [4].

The causes of IBS remain poorly understood, and include altered gut motor function, visceral hypersensitivity, abnormal gas handling, alterations in the central nervous system, mild inflammation, disturbances in serotonin handling and genetic factors (reviewed in [5]). Evidence has been mounting in the past decade that alterations in the gastrointestinal microbiota may also play a role in IBS. An early study showed that patients with IBS-D excreted abnormally high levels of hydrogen compared to healthy individuals, suggesting that there was abnormal bacterial fermentation of ingested foods [6]. An exclusion diet reduced this. More recently the gastrointestinal microbiota of IBS patients have been compared with those from healthy subjects by bacterial culture or molecular approaches [reviewed in [7,8]. Many studies have found alterations in the numbers of bacterial species present, or in the abundance of particular bacterial species. The findings vary, but a common feature seems to be a reduction in the number of Bifidobacteria and increased numbers of Firmicutes and Enterobacteriaceae in samples from IBS patients compared to control samples.

IBS has also been linked to small intestinal bacterial overgrowth, [reviewed in [7,9,10], although this is currently somewhat controversial [8]. In SIBO, bacterial numbers can rise from normal levels of 10^0-4^ colony forming units (cfu) of bacteria/ml in the terminal jejunum, 10^0-5^ cfu/ml in the proximal ileum and 10^5-8^ cfu/ml in the terminal ileum [11,12] to 10^11^ cfu/ml [9]. SIBO is currently defined as the presence of ≥ 1 × 10^5^ cfu/ml of colonic bacteria in the jejunum [13,14]. In contrast to the normal situation in which digestion and absorption of food is complete before the bolus reaches bacteria in the colon, in patients with SIBO, ingested food comes into contact with bacteria in the jejunum and ileum, and is fermented to produce gas, which has been visualised in the small intestine by abdominal radiography [15]. SIBO is typically diagnosed by giving patients a drink containing a poorly digestible sugar such as lactulose, and analysing their breath gases at intervals (reviewed in [16,17]). Glucose can also be used. The main gases excreted are hydrogen, which is associated with IBS-D, and methane, which is associated with IBS-C [18-21]. In one study, 78% of IBS patients tested positive for SIBO using the lactulose hydrogen breath test (LHBT) [22].

There is currently some controversy surrounding the use of lactulose hydrogen breath tests to diagnose SIBO and, consequently, the role of SIBO in IBS. Early breath tests diagnosed SIBO if hydrogen was excreted in two bursts, corresponding to bacterial fermentation in the small intestine, and in the colon. Later, hydrogen excretion within 90 minutes of lactulose ingestion was taken as diagnostic of SIBO. However, a recent study has combined LHBTs with scintigraphy to follow the movement of a radiolabelled tracer through the gastrointestinal tract [23]. Hydrogen excretion within 90 minutes of lactulose ingestion was found to correlate with fast oro-caecal transit rather than SIBO [23], and it has been suggested that glucose would be a better fermentation substrate since it is absorbed before it reaches the colon [17,21,24]. Using the ability to culture 1 × 10^5^ colonic bacteria from duodenal or jejunal aspirates as the gold standard for SIBO diagnosis, SIBO was diagnosed in 4% of IBS patients and healthy control subjects in one study [14] and in 10.9% of IBS patients in another [25]. Modestly raised bacterial counts (≥ 1 × 10^3^ cfu/ml) were found in 43% of IBS patients compared to 12% of healthy controls in the first study [14] and 37% of IBS patients in the second [25], suggesting that perhaps a more modest overgrowth of bacteria than previously thought might contribute to IBS. Of note, lipopolysaccharide from Gram-negative bacteria speeds up gastrointestinal transit [26], raising the possibility that modest numbers of bacteria in the small intestine could speed up transit of lactulose (and chyme) to the colon, and result in a positive LHBT (and IBS) without fermenting the lactulose themselves.

One Gram-negative bacterium that has been implicated in IBS and SIBO is *Escherichia coli.* An early study found *E. coli* throughout the gastrointestinal tract of patients with SIBO [27]. *E. coli* was present in jejunal and duodenal aspirates from patients with IBS and SIBO [14,25], in addition to *Klebsiella* and *Enterococcus* species. *E. coli* was found to be more prevalent in the mucosal microbiota of IBS patients than those of healthy control subjects when biopsy specimens were labelled with fluorescent in situ hybridisation probes [28], and enteroaggregative strains of *E. coli* were present in higher numbers in faecal samples from IBS patients than those from healthy individuals [29].

There are currently various medicines available to treat IBS, including fiber, antispasmodic agents, and antidepressants to modulate pain perception [30]. Tricyclic antidepressants block diarrhoea whereas serotonin reuptake inhibitors can benefit IBS-C patients by stimulating gastrointestinal motility [30]. In agreement with the idea that alterations in the gastrointestinal microbiota are involved in IBS, there has been considerable success with treatments that reverse this. Clinical trials have shown that antibiotics can be effective in treating IBS [31,32]. Successful eradication of SIBO, and reversal of the symptoms of IBS have been achieved by treating IBS patients with antibiotics such as metronidazole [33], neomycin [18], the non-absorbable antibiotic rifaximin (reviewed in [34]), a combination of rifaximin and ciprofloxacin [35], or a combination of rifaximin and neomycin for IBS-C patients who produce methane [36]. Reduction of hydrogen or methane excretion was linked to improvement in gastrointestinal symptoms and rifaximin was particularly effective in treating wind and bloating. Currently, NICE guidelines do not mention the use of antibiotics for the treatment of IBS in the UK [37]. SIBO can return after treatment, however [38] and there is concern that widespread and prolonged treatment with antibiotics could lead to the emergence of antibiotic resistant bacterial strains [39]. Indeed rifaximin can be used to treat infections with *Clostridium difficile*[40], but rifaximin-resistant strains have been isolated [41], and the fact that some IBS patients are carriers of *C. difficile*[42] raises the possibility that widespread treatment of IBS patients with rifaximin could lead to the production of more rifaximin-resistant strains of *C. difficile.*

In contrast, the use of probiotics aims to increase the number of beneficial bacteria. Probiotics are live microorganisms that, when administered in sufficient numbers, reduce visceral hypersensitivity, improve gastrointestinal dysmotility and epithelial integrity, improve immune function, and modulate the gut microbiota [7,8]. Two recent systematic reviews showed that probiotics had a moderate therapeutic benefit in improving IBS symptoms [43,44]. Probiotics reduced the pain of IBS and this was statistically significant; there was also a significant reduction in flatulence and a trend towards reduction in bloating [43]. In particular, lactic acid bacteria were shown to be useful, although the therapeutic benefit stopped when administration of the probiotics was terminated [44]. Related to this, prebiotics have also been shown to be of modest benefit in treating IBS – these are nondigestible dietary supplements that increase the growth of beneficial bacteria. One clinical trial showed that ingestion of galactooligosaccharide stimulated the growth of bifidobacteria and alleviated IBS symptoms [45]. Finally, faecal transplantation has been successfully used to modulate the gastrointestinal microbiota in a small number of IBS patients (reviewed in [46]). This has the advantage that the entire community of colonic bacteria is transplanted, rather than the one or two bacterial species that might be present in a probiotic preparation.

An alternative approach to the treatment of IBS is the use of dietary modifications to reduce the amount of fermentable substrates available to the gastrointestinal microbiota. Early work pioneered the use of an exclusion diet consisting of one meat, one fruit and distilled or spring water for a week. If the patient’s IBS symptoms resolved, foods were reintroduced one at a time and any resulting IBS symptoms noted [47]. Two thirds of the patients reported resolution of their symptoms on the exclusion diet, and wheat, corn, dairy products, coffee, tea and citrus fruits were found to provoke IBS symptoms, even in a double blind food challenge. A more relaxed exclusion diet consisting of fish and meat (apart from beef) and rice, and lacking dairy products, citrus fruits, yeast, tap water and caffeinated drinks, reduced hydrogen excretion by IBS patients in addition to resolving their symptoms [6]. This exclusion diet has been described in more detail [48]. These and other exclusion diets have been found to be useful under medical supervision by NICE [37], which also recommended that it may be helpful for IBS patients to limit both their fibre intake, and to eat no more than three portions of fruit per day. A recent approach to dietary manipulation has rationalised knowledge of the foods that commonly provoke IBS symptoms with an understanding of their chemical composition, namely the adoption of a diet low in FODMAPs (fermentable oligosaccharides, disaccharides, monosaccharides and polyols) [49]. This involves avoiding 1) fruits that are high in fructose, 2) dairy products apart from butter and hard cheese, because they contain lactose, 3) vegetables, legumes and cereals (including wheat) that contain the oligosaccharides fructans or galactans and 4) fruits, vegetables and artificial sweeteners that contain polyols [49]. Restriction of fructose and fructans led to an improvement in gastrointestinal symptoms in three out of four IBS patients with fructose malabsorption [50]. When other patients who had attained remission of their IBS symptoms for 3 – 36 months on a low FODMAPs diet were rechallenged with fructose or fructans in a placebo-controlled, double blind clinical trial, their symptoms returned, indicating that the fructose and fructans could provoke IBS symptoms [51]. In another double blind trial by the same group (in Australia), IBS patients on a high FODMAPs diet excreted more hydrogen and experienced more gastrointestinal symptoms than patients on a low FODMAPs diet [52]. More recently, a British group has compared the efficacy of a low FODMAPs diet in reducing IBS symptoms with the standard dietary guidelines recommended by NICE: more of the patients on the low FODMAPs diet reported an improvement in their symptoms than the patients following the NICE dietary guidelines [53], this was especially true for wind and bloating.

Herbal medicines are also used to treat IBS, both in mainstream medicine, and in complementary and alternative medicines from different traditions; these have been discussed in several excellent reviews [54-56]. Peppermint (*Mentha piperita*) oil has been recommended for the treatment of IBS by NICE guidelines [37] and is widely prescribed in the form of enteric-coated peppermint oil tablets; it has antispasmodic activity [57,58]. Grigoleit and Grigoleit performed a meta-analysis of sixteen placebo-controlled clinical trials studying the use of enteric-coated peppermint oil capsules to treat IBS, and found that the overall success rate of peppermint oil capsules was 58% compared to 29% for placebo [59]. They concluded that “peppermint oil…may the drug of first choice in IBS patients with non-serious constipation or diarrhoea to alleviate general symptoms and to improve quality of life e.g. pain or bloating”. A recent meta-analysis of four clinical trials confirmed this view [60]. In fact peppermint oil, in the form of a preparation called Peppermint water BP1973, has long been used to treat dyspepsia, flatulence and stomach cramps, and has just been awarded a traditional herbal registration certificate [61]. Another herbal preparation that has shown promise in the treatment of IBS is Iberogast® (also called STW-5), a liquid formulation of nine different herbs, namely bitter candytuft, or clown’s mustard plant (*Iberis amara*), German chamomile *(Matricaria recutita*) flowers, angelica (*Angelica archangelica*) root, caraway (*Carum carvi*) fruit, lemon balm (*Melissa officinalis*) leaves, greater celandine (*Chelidonium majus*) aerial parts, liquorice (*Glycyrrhiza glabra*) root, milk thistle *(Silybum marianum* L) and peppermint oil [62]. In a randomised, double-blind, placebo-controlled trial, STW 5 (Iberogast®) reduced abdominal pain, problems with bowel habit and flatulence in IBS patients [63]. Iberogast® is also effective against functional dyspepsia [64] and is widely prescribed in Germany with more than a million prescriptions being written for it in Germany in 2002 [65]. Iberogast® has been shown to have antispasmodic, anti-inflammatory and antioxidant activity, and also acts as a secretagogue (reviewed in [55,62]). A similar mixed herbal extract, called carmint, which consists of herbal extracts of lemon balm, spearmint (*Mentha spicata*) and coriander (*Coriandrum sativum*), reduced abdominal pain and bloating in IBS patients in a clinical trial [66]. IBS-D patients were prescribed loperamide, and IBS-C patients were prescribed psyllium, plus either carmint or placebo; there was a significant reduction in symptoms in the IBS patients taking carmint compared to those taking the placebo after eight weeks.

Herbs have traditionally been used to treat bacterial infections [67], for instance lavender (*Lavandula angustifolia*) oil and tincture were used to treat wounds before the First World War. Many culinary herbs have been reported to possess antibacterial properties [67,68], as have the essential oils of these herbs [69]. Many herbs, for instance fennel (*Foeniculum vulgare*), lavender, peppermint, rosemary (*Rosmarinus officinalis*) and sage (*Salvia officinalis*), have been used traditionally as digestives, aiding digestion or reducing flatulence [67,68,70]. Our hypothesis is that the digestive properties of a particular herb may be linked, at least in part, to the herb’s antibacterial action. Essential oils and herbal extracts have an advantage over conventional antibiotics since they may contain several antibacterial compounds that act in different ways, so that it would be more difficult for bacteria to develop resistance. For instance lemon grass (*Cymbopogon citratus*) essential oil contains at least sixteen compounds [71] and successfully inhibited the growth of *Helicobacter pylori* over many bacterial generations, whereas antibiotic resistant *H. pylori* emerged after ten passages on plates containing only the antibiotic clarithromycin [72].

NICE guidelines recommend that further research should be conducted to study the possibility of using herbal medicines to treat IBS. With this in mind, in order to increase our understanding of the mechanism of action of herbal medicines that have been shown to be beneficial in treating IBS, and to identify other essential oils or extracts that would be useful candidates for clinical trials, we have conducted a preliminary study *in vitro* using a non-pathological strain of *E. coli.* We have compared the antibacterial activity of essential oils of a range of herbs that have been used traditionally as digestives, in three separate assays. We found that the essential oils with the most potent antibacterial activity in the three assays were those of coriander seed, lemon balm and peppermint. Interestingly, essential oils or extracts of coriander, lemon balm and peppermint are all present in herbal medicines that have been validated for use in the treatment of IBS by at least one clinical trial (without their mechanism of action being ascribed to an antibacterial effect). We identified the compounds present in the coriander seed, lemon balm and peppermint essential oils that we had used, by thermal desorption gas chromatography mass spectrometry. Finally we tested the antibacterial activity of extracts of coriander, lemon balm and spearmint leaves that had been made with various solvents to determine whether ethanolic tinctures of these herbs (which have been used instead of essential oils in some of the IBS medicines) would have antibacterial activity. This has allowed us to propose a new mechanism of action for these herbal medicines, and suggest some other herbs/essential oils that could be tested in further clinical trials.

## Methods

### Materials

*E. coli* strain DH5α, was obtained from Dr. Geertje van Keulen (Swansea University). All essential oils (listed in Table 1) were obtained from Amphora Aromatics, Bristol, UK. Coriander plants were obtained from Tesco Supermarket, Swansea, lemon balm plants were from the Swansea City Council Botanic Gardens shop and spearmint plants were obtained from the Homebase superstore in Swansea. All chemicals were obtained from Sigma Chemical Company (Poole, Dorset) unless otherwise stated.

**Table 1:** Summary of results of antibacterial activity of essential oils

**Vernacular name**	**Botanical name**	**Activity in disc diffusion assay**	**Activity in turbido-metric assay**	**Activity in zone of clearance assay**
Coriander seed	*Coriandrum sativum*	++	+++	+++
		(0.0053)	(5.82 × 10^-5^)	(0.0002)
Fennel	*Foeniculum vulgare*	+	+++	+
		(0.0003)	(8.01 × 10^-6^)	(0.0027)
Grapeseed	*Vitis vinifera*	-	-	-
			(0.3216)	
Lavender	*Lavendula angustifolia*	+	++	+
		(0.0039)	(0.0031)	(0.0011)
Lemon balm	*Melissa officinalis*	++	++	+++
		(0.0041)	(0.0008)	(0.0014)
Lemon grass	*Cymbopogon citratus*	++	++	+
		(0.0004)	(0.0030)	(0.0003)
Mandarin	*Citrus reticulata*	++	+++	+
		(0.0092)	(3.39 × 10^-5^)	(0.0484)
Neem	*Azadirachta indica*	-	ND	-
Peppermint	*Mentha piperita*	++	+++	+++
		(0.0390)	(3.14 × 10^-5^)	(0.0000)
Pine	*Pinus sylvestris*	+++	++	++
		(0.0027)	(0.0003)	(0.0001)
Rosemary	*Rosmarinus officinalis*	+	++	+
		(0.0000)	(0.0085)	(0.0036)
Sage	*Salvia lavendulifolia*	+	+++	+
		(0.0022)	(3.39 × 10^-6^)	(0.0021)
Tea tree	*Melaleuca alternifolia*	+++	+++	+++
		(0.0079)	(5.83 × 10^-6^)	(0.0000)
Thyme	*Thymus vulgaris*	+++	+++	+
		(0.0020)	(5.84 × 10^-5^)	(0.0193)
Ylang ylang	*Cananga odorata* var. *genuina*	+/-	+	-
		(0.0075)	(0.0477)	

Crosses denote the magnitude of the antibacterial activity; the numbers in brackets are the P values obtained from t tests comparing the size of the halos produced with the essential oils with those produced by grapeseed oil for the disc diffusion and zone of clearance assays, and from comparing the A_600_ of the turbidometric assay in the presence of essential oil after 6 hours with that lacking essential oil.

### Disc diffusion assay: growth inhibition

A modification of the Kirby-Bauer disc diffusion assay was used [73]. *E. coli* bacteria were grown overnight in Luria Bertani medium [74] at 37°C with shaking at 150 rpm for 16 h and 100 μl of the resulting culture were spread onto LB agar plates [74] as a lawn. Sterile 7 mm glass fibre discs (Whatmann GF-C, Sigma, Poole, Dorset, UK) were immediately placed on the surface of the bacterial plates and essential oil (10 μl) was added to each disc. Grapeseed (*Vitis vinifera* seed) oil was included as a negative control, since it was not expected to exhibit any antibacterial activity [75]. Following growth at 37°C for 24 h, the zones of inhibition (halos) were measured with a ruler to an accuracy of 0.5 mm. An absence of antibacterial activity would produce a halo of 7 mm diameter, the size of the glass fibre discs. Disc diffusion assays were also carried out using 10 μl of the following antibiotic solutions in order to compare the antibacterial activities of some essential oils with those of the antibiotics: ampicillin (100 mg/ml in water), neomycin (10 mg/ml in water), and rifaximin (100 mg/ml in methanol), with disc diffusion assays also being carried out with water or methanol for comparison.

### Disc diffusion assay: zone of clearance

This assay was adapted from that of Bexfield *et al.*[76]. *E. coli* bacterial overnight culture (100 μl) was spread aseptically onto LB agar plates, and grown overnight at 37°C for 16 h. Sterile 7 mm glass fibre discs containing 10 μl essential oil were added to each disc. Grapeseed oil was used as a control. The plates were grown at 37°C for a further 2 days and the diameters of the zones of clearance were measured with a ruler to an accuracy of 0.5 mm.

### Turbidometric assay

*E. coli* DH5α were grown in LB medium for 16 h overnight and the concentration of bacteria in the overnight culture was determined by measuring A_600_[77]. Essential oil (100 μl) was added to 102 ml LB medium in a 500 ml conical flask. The flask was shaken to mix the contents and 2 ml of the liquid were removed to cuvettes for use as blanks. Bacterial overnight culture containing 1 × 10^8^*E. coli* cfu was added to the flask, which was shaken to mix the contents. A sample was removed to a cuvette and the A_600_ was measured against the blank. The bacteria were grown at 37°C with shaking at 150 rpm and the A_600_ was measured after 1 h and every 20 min thereafter for a further 5 h by sampling from the flask. Controls were performed, either lacking essential oil, or containing 100 μl grapeseed oil.

### GC/MS of essential oils

The chemical composition of the oils was evaluated by injecting samples of dilute oil onto sorbent tubes, then extracting with thermal desorption for analysis using gas chromatography with mass spectrometry (GC/MS).

Oils were diluted to 1 part in 1000 in HPLC grade methanol and injected in 10 μL quantities into sorbent tubes (6.4 mm diameter stainless steel containing Tenax TA® sorbent). A stream of helium was used to remove the methanol over 3 minutes at a flow rate of 30 ml/min and at room temperature. Sample tubes containing the oils were loaded into an Ultra unit (Markes International, Llantrisant, UK) for automated processing via a Unity thermal desorption unit and fed with inert helium at 10 psi to desorb the tubes in a 30 ml/min stream of inert helium at 300°C for 5 minutes. To further concentrate, the flow was driven onto a cold trap (U-T11GPC, general purpose graphitized carbon C4/5-C30/32) set at -10°C. The trap was then desorbed at 300°C for 3 minutes with 50 ml of the sample stream vented and the remainder injected into the GC (Agilent Technologies 6890 N gas chromatograph). The VOCs were separated using a capillary column (30 m × 0.25 mm id, HP-5MS film thickness 0.25 μm). The column temperature was initially set at 40°C and then increased steadily to 200°C at a rate of 5°C/min. Mass spectrometry (Agilent Technologies 5973 network mass selective detector) was used in electronic ionisation mode and mass spectral data obtained in the SCAN mode with a mass range m/z 40 - 550. Each tube was run twice to prevent carry-over of volatiles onto the cold trap in subsequent samples and to check for VOCs inherent in the system. Three samples were taken for each type of oil.

Automated peak detection and baseline correction was used to calculate peak area and retention time (RT) for each compound. Using an automated library search function in the Chemstation GC/MS software (Agilent Technologies), VOCs were tentatively identified using the NIST 98 mass spectral library (The National Institute of Standards and Technology) at the apex of each peak and utilising probability based matching (PBM). The list of compounds generated was re-checked using forward-search matching and compared with the literature. The consistency of the mass response across the retention time range was checked by injecting aliquots of toluene, xylene and dodecane directly into sorbent tubes (1 part in 10,000 in methanol), and measuring the area under the curve. These compounds gave very similar mass responses for given concentrations, indicating that the area under the curve for each compound could be used to approximately indicate the abundance level.

The compounds that make up essential oils are often present in a range of optical and structural isomers with similar mass spectra and sometimes differing retention times. It is therefore difficult to precisely identify all compounds [78]. The separation and analysis methods employed do not allow identification of optical isomers, or all structural isomers, so while the compounds present will probably be specific optical and structural isomers, compound identification has been limited to generic, rather than specific, isomeric structures.

### Preparation of plant extracts

Extracts were made from freeze dried coriander, lemon balm and spearmint leaves with various solvents using a modification of the methods of Wong and Kitts [79] for coriander and lemon balm or Lopez *et al.*[80] for mint. In each case, freeze dried leaf material was ground or chopped with a razor blade, resuspended in 10 ml solvent and left to macerate at room temperature for 18 or 24 h. The extracts were filtered through filter paper (Whatmann No. 1, Fisher Scientific. Loughborough, UK) and either used neat, or dried in a rotary evaporator set to 50°C and resuspended in the appropriate solvent. Extracts were stored at –20°C and equilibrated to room temperature before use. The extracts were tested in disc diffusion assays. Negative controls consisted of the solvent that had been used to prepare the extract, in each case, whereas the appropriate essential oil was used as the positive control.

### Statistics

The null hypothesis states that there is no difference between the diameter of the halos in the disc diffusion assays or the A_600_ values in the turbidometric assays, and those of the controls. Two-tailed independent samples t tests were carried out to determine the probability that the diameter of the halos in the disc diffusion asays differed from those of the controls by chance. The results of the turbidometric assays were analysed by a one-way ANOVA and post-hoc test using SPSS.

## Results

### Disc diffusion assays to examine the antibacterial activity of essential oils against *E. coli*

**Figure 1:**
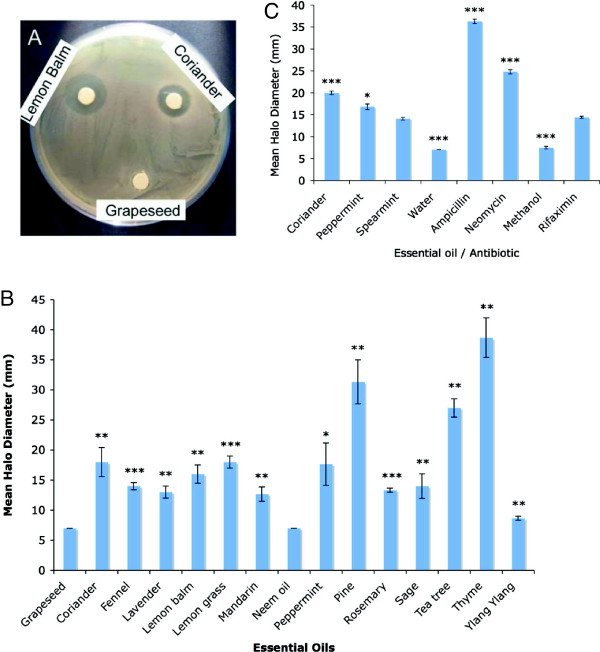
**Antibacterial activity of essential oils in disc diffusion assays. A)** Example of disc diffusion assay plate showing the halos in the bacterial lawn resulting from the antibacterial activity of lemon balm and coriander oils against *E. coli* DH5α. **B)** Graph of mean halo diameters from three disc diffusion assays showing the effect of essential oils on the growth of *E. coli,* +/- SEM. **C)** Graph of mean halo diameters from four replicates of a single disc diffusion assay showing the effect of essential oils or antibiotics on the growth of *E. coli,* +/- SEM. t tests were carried out to determine whether the halos produced by rifaximin were significantly different from the solvents, essential oils or other antibiotics. Significance levels obtained from two-tailed t tests are denoted by stars: * = significant (P < 0.05); ** = highly significant (P < 0.01); *** = very highly significant (P < 0.001).

Plant essential oils were tested for their antibacterial activity against *E. coli* DH5α by disc diffusion assays (see Figure 1). The largest halo diameters were produced by pine (*Pinus sylvestris*), thyme (*Thymus officinalis*) and tea tree (*Melaleuca alternifolia*) oils, with mean halo diameters of 38.7 +/- 3.3 mm for thyme, 31.3 +/- 3.7 mm for pine and 27 +/- 1.5 mm for tea tree, against a mean halo diameter of 7 +/- 0 mm for the grapeseed oil negative control. The results were highly significantly different from the control in each case (P < 0.01). This demonstrates that these oils have strong antibacterial activity towards *E. coli*.

Antibacterial activity against *E. coli* was recorded at a more moderate, but still statistically significant level with coriander seed (mean halo diameter of 18 +/- 2.4 mm), lemon grass (18 +/- 1 mm), peppermint (17.7 +/- 3.5 mm), lemon balm (16 +/- 1.5 mm), fennel (14 +/- 0.6 mm), sage (14 +/- 2 mm), rosemary (13.3 +/- 0.3 mm), lavender (13 +/- 1 mm) and mandarin (*Citrus reticulata*) (12.7 +/- 1.2 mm). The antibacterial activity of peppermint essential oil was statistically significantly different from that of the grapeseed oil control whereas the activities of coriander seed, lemon balm, sage, lavender and mandarin essential oils were highly significantly different and those of lemon grass, fennel and rosemary very highly significantly different from that of the control. Ylang ylang (*Cananga odorata* var. *genuina*) demonstrated only a very weak antibacterial activity against *E. coli* (mean halo diameter = 8.7 +/- 0.3 mm), which was, however, highly statistically significant. Like grapeseed oil, neem (*Azadirachta indica*) oil did not exhibit any antibacterial effect in this assay.

Disc diffusion assays were also conducted to compare the diameter of the halos produced by the antibiotic rifaximin (which has been shown to reverse SIBO in clinical trials) with halos produced by two other antibiotics: neomycin (used to treat SIBO and IBS clinically) and ampicillin (used to inhibit the growth of *E. coli* DH5a in the laboratory), and essential oils of peppermint, coriander seed and spearmint. The results are shown in Figure 1C. As expected, the mean diameter of the halos produced by rifaximin in the disc diffusion assays was larger and very highly significantly different from that produced by the methanol control, demonstrating that the rifaximin solution had antibacterial activity against *E. coli* DH5α which was not due to the methanol that was used to dissolve the rifaximin. The halos produced by neomycin and ampicillin were larger than those produced by rifaximin, showing that these antibiotics are more potent at killing/inhibiting the growth of *E. coli* in the disc diffusion assay than rifaximin, and the results were very highly significant. Interestingly, there was no significant difference between the diameter of the halos produced by rifaximin with those produced by spearmint essential oil, suggesting that, at least in this assay, spearmint essential oil is just as potent as rifaximin. Moreover, the halos produced by peppermint and coriander seed essential oils were larger and either significantly different, or very highly significantly different from the rifaximin in each case, demonstrating that peppermint and coriander seed essential oils have a larger antibacterial activity against *E. coli* DH5α in this assay than rifaximin.

### Turbidometric assays to examine the antibacterial activity of essential oils against *E. coli*

**Figure 2:**
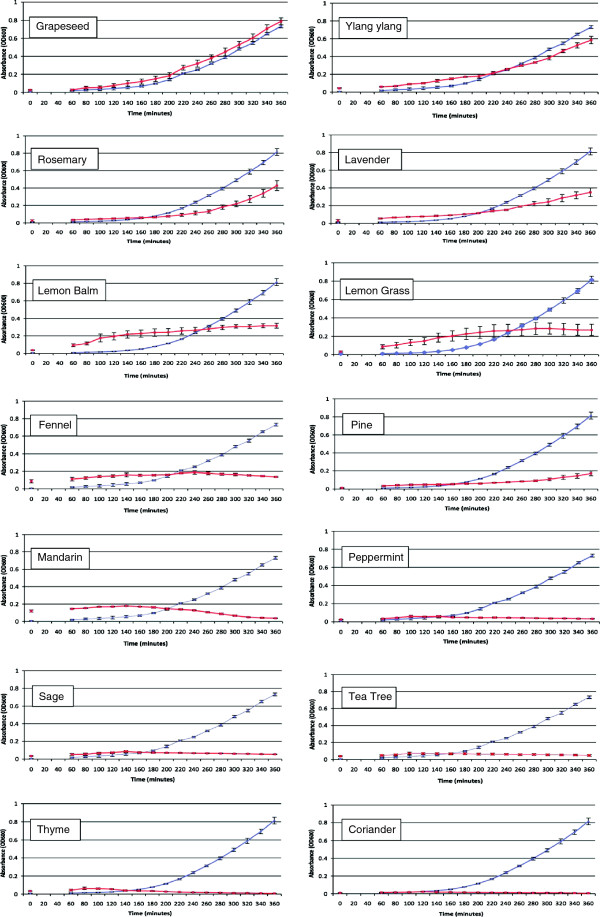
**Antibacterial activity of essential oils in turbidometric assays.** Graphs showing the results of turbidometric assays to examine the effect of plant essential oils on the growth of *E. coli* in liquid culture. Data are the mean of three experiments +/- SEM.

The antibacterial activities of the various essential oils against *E. coli* were also examined using turbidometric assays since this method is said to be more sensitive than the disc diffusion assay [81]. It can also reveal whether the oil slows down the growth of bacteria, or inhibits growth altogether. The results are shown in Figure 2. The Absorbance_600_ values obtained after 360 minutes, corresponding to bacterial growth were analysed by one way ANOVA. There were significant differences in variance detected between the mean absorbance values at t = 360 mins, F_14, 30_ = 3253.911, p < 0.0001. Post hoc extraction (Least Significant Difference method) revealed that there were significant differences between the standard and the samples containing the essential oils, with the exception of grapeseed oil (p < 0.05), i.e. all of the essential oils inhibited the growth of *E. coli* DH5α in the turbidometric assay, except for grapeseed oil.

In fact grapeseed oil produced a small and reproducible stimulation of the growth of *E. coli* compared to the standard lacking essential oil in this assay (Figure 2), suggesting that it is acting as a prebiotic. The other oils all inhibited the growth of *E. coli*. Ylang ylang oil inhibited *E. coli* growth by a small amount whereas rosemary, lavender, lemon balm, lemon grass, fennel and pine inhibited *E. coli* growth more strongly. The curves for samples containing these oils were exponential, indicating that there was some bacterial growth, albeit less than in the control. Mandarin, peppermint, sage, tea tree, thyme and coriander seed essential oils almost completely inhibited the growth of *E. coli* in the turbidometric assay, the growth curves were flat.

Samples containing lemon balm, lemon grass or mandarin essential oils had a high OD_600_, even at the start of the bacterial incubation, although the absorbances did not rise very steeply after that and, in the case of mandarin oil, fell. This should not have happened and cannot be attributed to light absorbance at 600 nm by coloured compounds in the oils, since the spectrophotometer was zeroed against LB blanks containing the appropriate oil. The effect was reproducible with certain oils, but the reason for it is not clear – perhaps some of the oils formed light scattering micelles.

### Zone of clearance assay

In order to investigate whether the essential oils could lyse *E. coli* that were already present rather than inhibiting their growth, zone of clearance assays were conducted, ie glass fibre discs containing essential oils were placed on pre-existing *E. coli* lawns, and halos were allowed to develop. This was important because treatments to target SIBO would need to kill bacteria that were already present in the small intestine, rather than simply inhibiting their growth.

**Figure 3:**
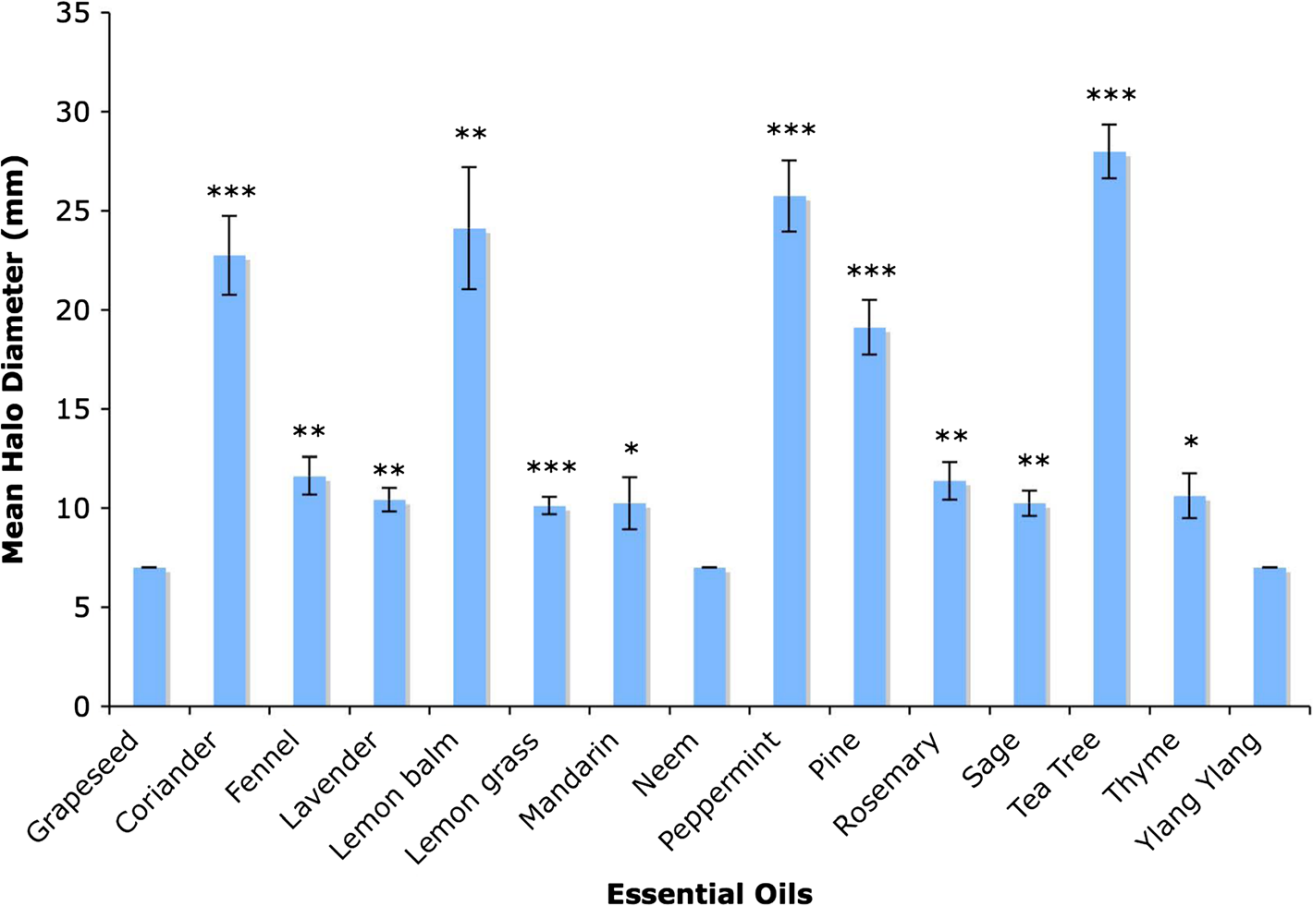
**Antibacterial activity of essential oils in zone of clearance assays.** Graph showing mean halo diameters +/- SEM obtained from four separate zone of clearance assays to determine the ability of plant essential oils to lyse *E. coli.* Significance levels obtained from two-tailed t tests are denoted by stars: * = Significant, P < 0.05; ** = highly significant, P < 0.01; *** = very highly significant, P < 0.001.

Coriander seed, lemon balm, peppermint, pine and tea tree oils exhibited strong antibacterial activity in the zone of clearance assay (see Figure 3), with mean halo diameters of 28.0 +/- 1.4 mm for tea tree, 25.8 +/- 1.8 mm for peppermint, 24.1 +/- 3.1 mm for lemon balm, 22.8 +/- 2.0 mm for coriander seed and 19.1 mm +/- 1.4 mm for pine essential oils, compared to a mean halo diameter of 7 +/- 0 mm for grapeseed oil. t tests revealed that the halo diameters for coriander seed, peppermint, pine and tea tree were very highly significantly different from those of the grapeseed oil control (P < 0.001) whereas the halo diameters for lemon balm were highly significantly different from those of the control (p < 0.01). Many of the other oils exhibited a small effect in the zone of clearance assay, namely fennel (mean halo diameter: 11.6 +/- 0.9), rosemary (11.4 +/- 0.9 mm), thyme (10.6 +/- 1.1 mm), lavender (10.4 +/- 0.6 mm), mandarin (10.3 +/- 1.3 mm), sage (10.3 +/- 0.6 mm) and lemon grass (10.1 +/- 0.4 mm). The halos for mandarin and thyme were significantly different from those of the grapeseed oil control whereas those for fennel, lavender, rosemary and sage were highly significantly different and the halos for lemon grass were very highly significantly different from the halos produced by the grapeseed oil control.

The results of the disc diffusion assays, the turbidometric assays and the zone of clearance assays have been summarised in Table 1. It can be seen that coriander seed, lemon balm, peppermint, pine and tea tree essential oils performed well in all three assays.

### Mass spectrometry of coriander seed, lemon balm and peppermint essential oils

Since coriander, lemon balm and peppermint are all culinary herbs and their essential oils are all on the list of food additives that are generally recognised as safe to ingest [82], thermal desorption gas chromatography mass spectrometry (GC/MS) was conducted on the coriander seed, lemon balm and peppermint essential oils used in this study to identify the compounds present, and to assess whether the oils were representative of their respective types.

**Figure 4:**
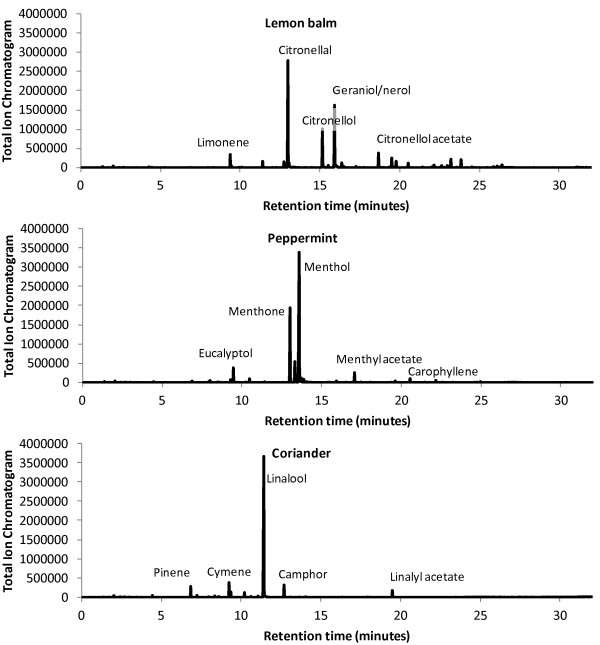
Chromatograms of lemon balm, peppermint and coriander oils.

Sample chromatograms are shown in Figure 4. Lists of the main compounds detected by GC/MS are tabulated for lemon balm, peppermint and coriander seed oils in Tables 2, 3 and 4 respectively. The approximate concentration is calculated as the mean of three samples of each essential oil. Compound identification is tentative and based on correlation with a mass spectra database. The compounds shown are those present in approximate concentrations of 1% or more and were primarily in the form of terpenes or terpinoids.

**Table 2:** Major compounds detected in lemon balm oil

**Compound (Tentative identification)**	**Approximate percentage mass**
Citronellal	29.79
Geraniol/Nerol	19.85
Citronellol	11.20
Citronellol acetate	4.07
Isopulegol	3.59
Limonene	3.23
Linalyl acetate	2.80
Unknown	2.62
Unknown	2.55
Unknown	1.87
Linalool	1.65
Camphene	1.44
Citral	1.42
Caryophyllene	1.35
Unknown	1.05

**Table 3:** Major compounds detected in peppermint oil

**Compound (Tentative identification)**	**Approximate percentage mass**
Menthol	52.33
Menthone	29.96
1,8-cineole (eucalyptol)	3.70
Menthyl acetate	2.96
Unknown	1.80
Caryophyllene	1.24

**Table 4:** Major compounds detected in coriander seed oil

**Compound (Tentative identification)**	**Approximate percentage mass**
Linalool	72.16
Cymene	5.58
Camphor	5.58
Pinene	3.97
Linalyl acetate	3.15
Limonene	2.12
Terpinene	1.86

The lemon balm oil primarily consisted of citronellal, geraniol/nerol (alcohol form of citral) and citronellol, with smaller amounts of citronellol acetate, isopulegol and limonene (see Table 2). It was unusual in containing only a low level of citral, which consists of a mixture of the two isomeric compounds geranial and neral, and is usually reported to be a major constituent of lemon balm oil [83-85], although lemon balm oils with only slightly higher levels of citral than ours have been documented [86]. Some of the lower concentration compounds gave similar library matches to one another despite being detected at different retention times. This suggests similar structures, which could not be adequately distinguished by the techniques used in the study, and these compounds have been labelled as “unknown”. The peppermint oil consisted primarily of menthol and menthone, with smaller amounts of eucalyptol (1,8-cineole), menthyl acetate and caryophyllene (see Table 3), which is in agreement with other published analyses of peppermint oil [87-89]. The coriander seed oil consisted primarily of linalool, with smaller amounts of cymene, camphor, pinene, linalyl acetate, limonene and terpinene (see Table 4). The compounds were similar to those reported elsewhere for coriander seed [90-93]. Again this suggests that the oil used in this study was representative of its type. Our analysis could not differentiate between isomers and many compounds present at lower concentrations were not adequately identified, and require further investigation.

### Disc diffusion assays to examine the effect of plant extracts on the growth of *E. coli*

The experiments in the earlier sections showed that the essential oils of coriander seed, lemon balm and peppermint exhibited antibacterial activity against *E. coli* in three separate assays, suggesting that they might reduce numbers of *E. coli* in the gastrointestinal tract, and thus be able to reverse intestinal dysbiosis and SIBO, and resolve the symptoms of IBS. In fact, a clinical trial has shown that a mixed herbal extract of coriander, lemon balm and spearmint (called carmint) reduced the symptoms of IBS after eight weeks [66]. Presumably carmint (said to contain “total extracts” of the three herbs [66]) is an ethanolic extract of coriander, lemon balm and spearmint leaves (and possibly stems), although this is not explicitly stated. The mechanism of action underlying carmint’s efficacy in treating IBS has not been described. In order to determine whether ethanolic extracts of coriander, lemon balm and spearmint leaves would have antibacterial activity, which could explain carmint’s efficacy, we made extracts of coriander, lemon balm and spearmint leaves using ethanol or various other solvents of differing polarity, and carried out disc diffusion assays. It should be noted that even if the extracts made with solvents other than ethanol had antibacterial activity, they would be useless therapeutically because the solvents would be toxic. The solvents were used as negative controls, since some would exhibit antibacterial activity in their own right, and the appropriate essential oil was used as the positive control.

### Coriander leaf extracts

**Figure 5:**
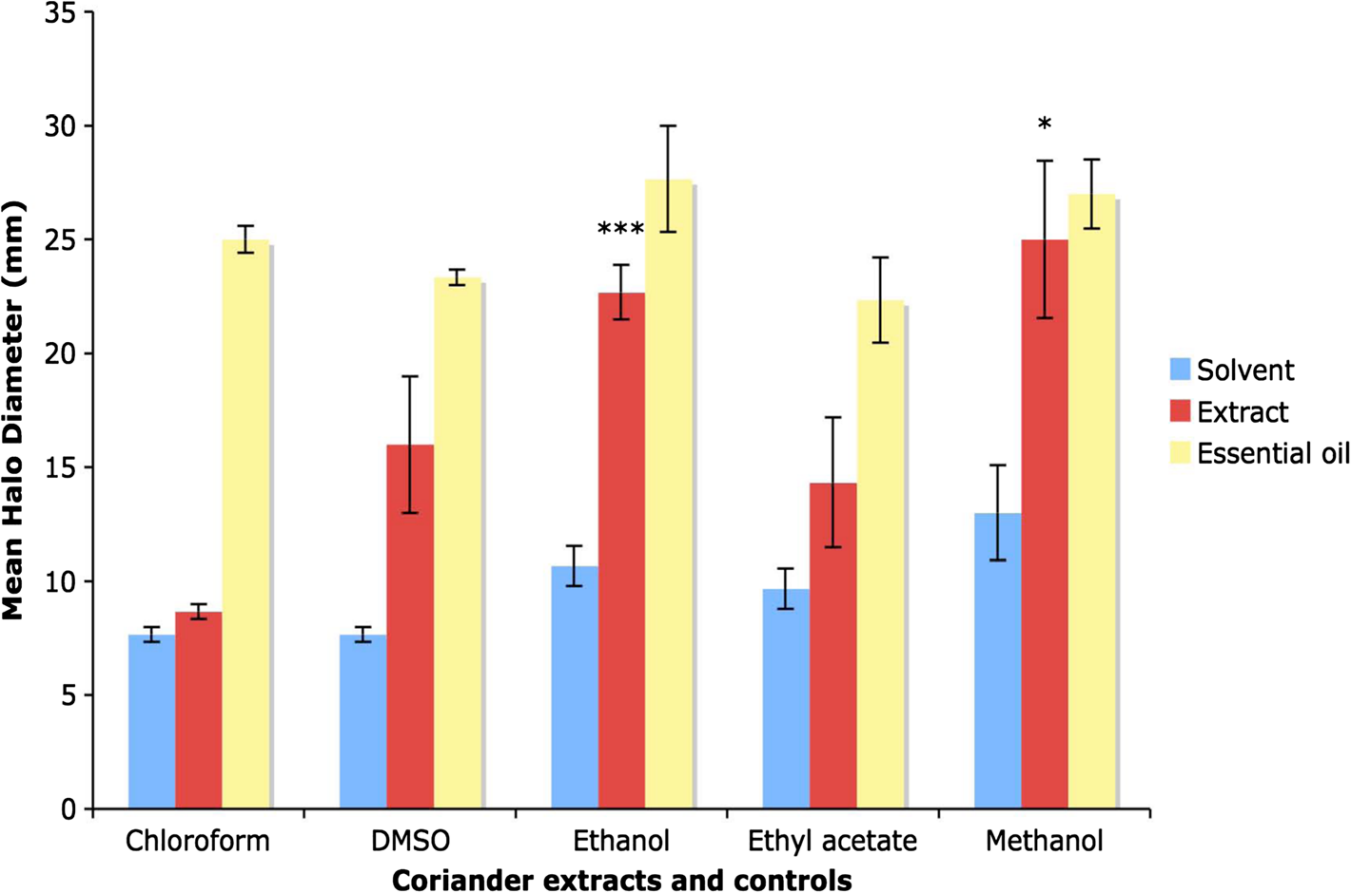
**Antibacterial activity of coriander extracts in disc diffusion assays.** Graph showing mean halo diameters +/- SEM obtained from three separate disc diffusion assays to determine the effect of coriander extracts made with various solvents on the growth of *E. coli.* Significance levels obtained from two-tailed t tests are denoted by stars: * = Significant, P < 0.05; ** = highly significant, P < 0.01; *** = very highly significant, P < 0.001. Significance levels for coriander essential oil were not determined.

The results for disc diffusion assays with coriander leaf extracts are shown in Figure 5. The mean halo diameter produced by the coriander extract that was made with chloroform is no larger than those produced by the chloroform control (8.7 +/- 0.3 mm versus 7.7 +/- 0.3 mm), thus the chloroform extract of coriander does not exhibit any antibacterial effects against *E. coli.* The coriander extract made with DMSO seemed to exhibit antibacterial activity, with a mean halo diameter of 16 +/- 3 mm, compared to 7.7 +/- 0.3 mm for the DMSO control, although the results fell short of significance. The ethanolic extract of coriander exhibited potent antibacterial activity, with a mean halo diameter of 22.7 +/- 1.2 mm versus 10.7 +/- 0.9 mm for the ethanol control, with the results being highly significant. The coriander extract made with ethyl acetate was not significantly different from the ethyl acetate control (mean halo diameters of 14.3 +/- 2.9 mm versus 9.7 +/- 0.9 mm). Finally, the methanolic extract of coriander also exhibited strong antibacterial activity, with a mean halo diameter of 25 +/- 3.5 mm versus 13 +/- 3.6 mm for the methanol control, with the results being statistically significant. Thus the ethanolic and methanolic extracts of coriander were the most potent at inhibiting the growth of *E. coli* in the disc diffusion assay, suggesting that the antibacterial compounds have been extracted into these polar solvents.

### Lemon balm extracts

**Figure 6:**
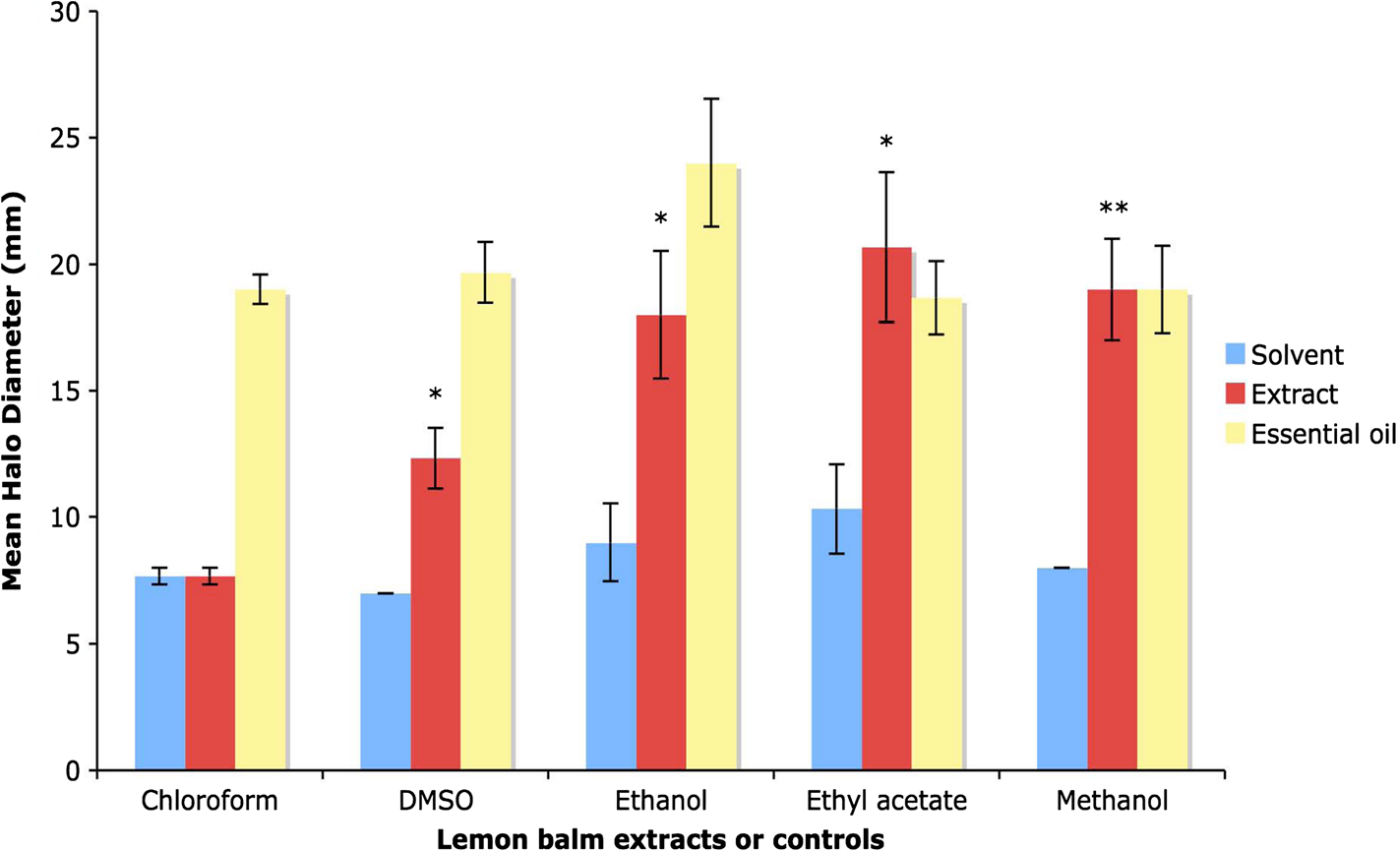
**Antibacterial activity of lemon balm extracts.** Graph showing mean halo diameters +/- SEM obtained from three separate disc diffusion experiments to determine the effect of lemon balm extracts made with various solvents on the growth of *E. coli.* Significance levels obtained from two-tailed t tests are denoted by stars: * = Significant, P < 0.05; ** = highly significant, P < 0.01; *** = very highly significant, P < 0.001. Significance levels for disc diffusion assays with lemon balm essential oil were not determined.

The results for disc diffusion assays with lemon balm extracts are shown in Figure 6. Lemon balm extract made with chloroform did not exhibit any antibacterial activity against *E. coli*, since the mean halo diameter was 7.7 +/- 0.3 mm for both the chloroform control and the chloroform extract. Extracts of lemon balm made with DMSO exhibited moderate antibacterial activity against *E. coli,* with a mean halo diameter of 12.3 +/- 1.2 mm versus 7 +/- 0 mm, with the results being statistically significant. More potent antibacterial activity was observed with lemon balm extracts made with ethanol, ethyl acetate and methanol, with mean halo diameters of 18 +/- 2.5 mm, 20.7 +/- 3 mm and 19 +/- 2 mm respectively versus 9 +/- 1.5, 10.3 +/- 1.8 and 8 +/- 0 mm respectively, for the solvent controls. The results for the extracts made with ethanol and ethyl acetate were statistically significant, and those for the methanolic extract were highly statistically significant. This suggests that the antibacterial compounds in lemon balm have been extracted into ethyl acetate, methanol and ethanol.

### Spearmint extracts

**Figure 7:**
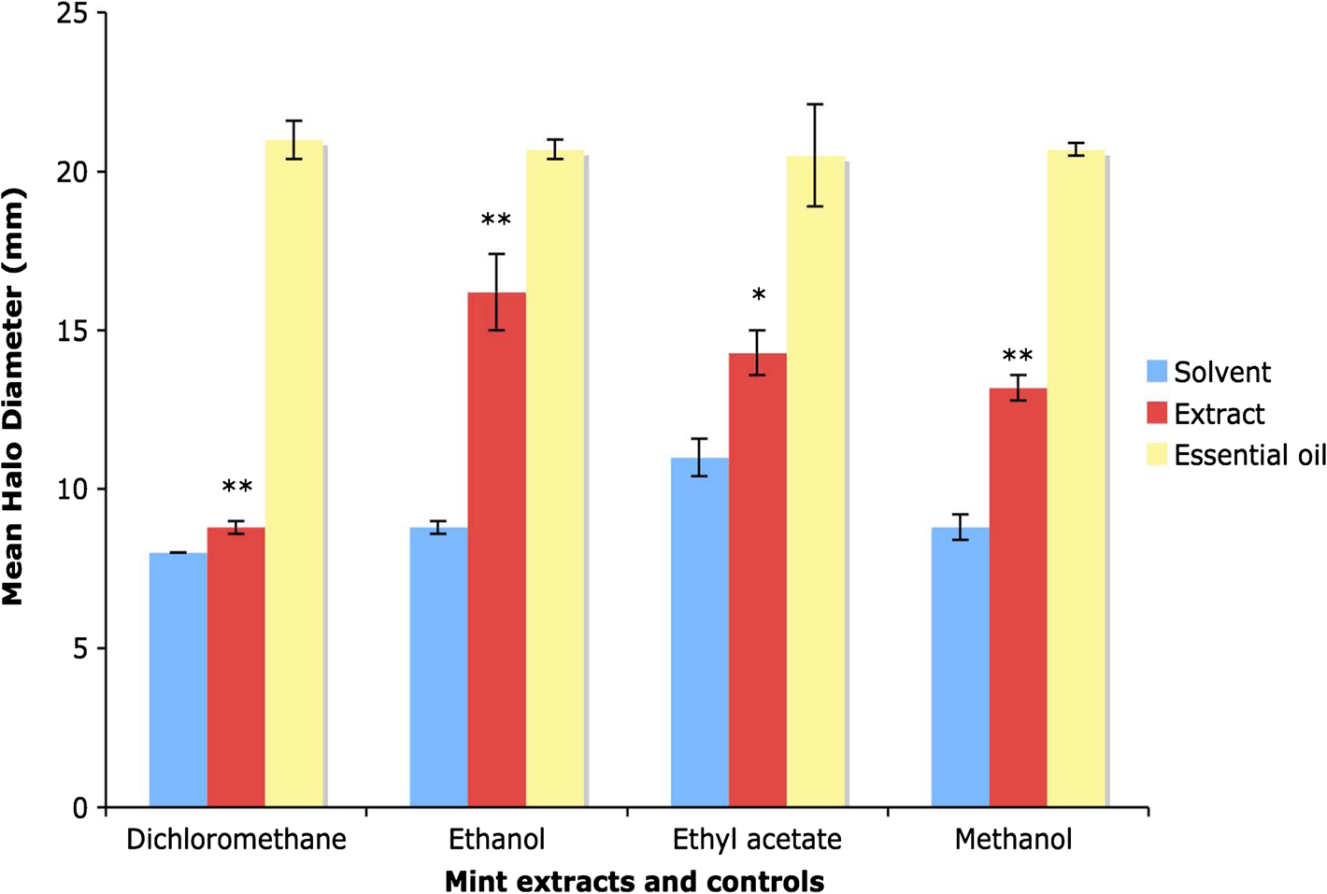
**Antibacterial activity of spearmint extracts.** Graph showing mean halo diameters +/- SEM from three disc diffusion assays to determine the effect of spearmint extracts made with various solvents on the growth of *E. coli.* Significance levels obtained from two-tailed t tests are denoted by stars: * = Significant, P < 0.05; ** = highly significant, P < 0.01; *** = very highly significant, P < 0.001. Significance levels for disc diffusion assays with peppermint essential oil were not determined.

The results for disc diffusion assays with spearmint extracts are shown in Figure 7. Spearmint extracts made with dichloromethane showed very little antibacterial effect against *E. coli,* producing halos with an average diameter of 8.8 +/- 0.2 mm compared to 8.0 +/- 0.0 mm for the dichloromethane control. The results were highly statistically significant, however. Spearmint extracts made with ethanol inhibited the growth of *E. coli*, producing halos of average diameter 16.2 +/- 1.2 mm versus 8.8 +/- 0.2 mm for the ethanol control, and the results were highly significant. Spearmint extracts made with ethyl acetate displayed antibacterial activity, producing halos of average diameter 14.3 +/- 0.7 mm versus average halo diameters of 11.0 +/- 0.6 mm for the solvent control; the results were statistically significant. Spearmint extracts prepared with methanol displayed a similar level of antibacterial activity, producing halos with a mean diameter of 13.2 + 0.4 mm versus 8.8 +/- 0.4 mm for the methanol control. The results were highly significant. This suggests that the antibacterial compounds in spearmint have been extracted into ethanol, ethyl acetate and methanol.

The results show that ethanolic extracts of coriander, lemon balm and spearmint leaves have antibacterial activity, and thus that the mixed herbal extract carmint (which contains extracts of coriander, lemon balm and spearmint) would be expected to have antibacterial activity, which would probably be responsible for its efficacy as a treatment for IBS.

## Discussion

The data above show that pine, tea tree and thyme oils all exhibited strong antibacterial activity against *E. coli* in the disc diffusion assay, with coriander seed, lemon balm, lemon grass, mandarin and peppermint essential oils having more moderate antibacterial activity. In each case the results were statistically significant. This agrees with reports in the literature since inhibition of the growth of different strains of *E. coli* has been demonstrated by coriander [94,95], fennel [94-97], lavender [94,98,99], lemon balm [98], lemon grass [94,98,100,101], mandarin [98,102], peppermint [94,96,97,99,103], pine [104], rosemary [96,97,105,106], sage [96,106], tea tree [94,107] thyme [96,107,108], and ylang ylang [94]. Most of these reports used disc diffusion assays or growth inhibition assays to determine the minimum inhibitory concentration of essential oils that would inhibit the growth of *E. coli*. In this present study, coriander seed, fennel, mandarin, peppermint, sage, tea tree and thyme essential oils all blocked the growth of *E. coli* in a turbidometric assay as well, whereas lavender, lemon balm, lemon grass, pine and rosemary oils exhibited strong antibacterial activity in this assay, at least for six hours, the approximate length of time that would elapse between doses of a theoretical IBS medicine, which would be taken before breakfast, lunch, dinner, and possibly bed time. Any one of these essential oils, or a mixture of them, would be a potential candidate for the reversal of SIBO and treatment of IBS. The advantages of using a mixture of essential oils rather than a single oil are that the larger number of compounds would act on a wider range of bacteria, the oils might act synergistically to have a stronger effect on any given bacterium (as has been documented for thyme and anise (*Pimpinella anisum*) oils [109]), and there would be a reduced chance of producing resistant bacteria.

Coriander seed, lemon balm, peppermint and tea tree essential oils demonstrated strong antibacterial effects against *E. coli* in a zone of clearance assay (and pine had a more moderate effect), indicating that they are bacteriocidal towards *E. coli*. Of these, coriander seed, lemon balm and peppermint essential oils are on the FDA list of food additives that are generally recognised as safe to ingest [82]. Indeed peppermint and coriander seed essential oils displayed higher antibacterial activity in a disc diffusion assay with *E. coli* DH5α than rifaximin, considered to be the antibiotic of choice for treating SIBO [34]. Thus coriander seed, lemon balm and peppermint essential oils would be very good candidates for the treatment of IBS, either singly or in combination, since they would be able to kill bacteria that were already present in the small intestine as SIBO, rather than just inhibiting their growth.

The compounds present in these three essential oils were identified by thermal desorption gas chromatography mass spectrometry; many have been reported to have antibacterial activity. Antibacterial activity against *E. coli* has been documented for linalool [98,100,108,110-113], cymene [114], camphor [100,110] and pinene [108,110], which are all present in coriander seed oil; citronellal [112], geraniol/nerol [98,100,108,111,112,115,116], citronellol [111], and isopulegol [117], which are present in lemon balm oil; limonene [102,108,110,113,118], which is present in both coriander seed and lemon balm oil; as well as caryophyllene [113], which is present in lemon balm and peppermint oil; and eucalyptol [100,110,111,119], menthol [100,110] and menthone [100,108,118], which are all present in peppermint oil. Citral, which is usually reported to be a major constituent of lemon balm oil, but was present only at a low level in the lemon balm oil that we used, has also been shown to have antibacterial activity against *E. coli*[98,100,108,112,115].

Lemon balm essential oil varies enormously in its chemical composition. This can be due to the use of different plant lines or cultivars [83-85], variation in plant growth conditions such as climate/soil [83] or salinity [86], harvesting at different points in the growing season, or in different years [84], different harvest cut height [120], use of either fresh or dried plant material to produce the essential oil [84] and storage of the essential oil, since citral levels decrease with storage time [84]. Citral levels have been shown to range between 3.1 +/- 0.35% and 5.4 +/- 0.12%, in essential oils from plants that were grown in the presence of salt and harvested at the full bloom stage [86], to between 10.86% and 64.56% in essential oils from plants that were not flowering [84], with Cosge *et al.*[85] reporting intermediate citral levels between 10.1% and 17.43% and higher citronellal levels between 36.62% and 43.78%. Patora *et al*. hypothesised that citral is converted to citronellal as the plant ages [84]. This highlights the need to conduct rigorous quality control analysis, if lemon balm (or any herbal product) is to be used medicinally. Further work is ongoing in our laboratory to investigate the variation in the chemical composition and antibacterial activity of different batches of lemon balm oil (and other essential oils) obtained from different companies, and to analyse the antibacterial activity of the individual compounds in the oils, with the aim of informing eventual quality control analysis of essential oils for use as medicines. We have several batches of lemon balm oil from the same company as the one that we used in this study, with very similar chemical composition, and several batches from another company with higher concentrations of citral.

For more in depth evaluation of essential oils, the use of more appropriate separation technologies such as chiral columns would aid the identification and separation of the various isomeric forms common in essential oils. Furthermore, the use of systems such as Kovats retention index would produce data independent of the chromatographic setting, which could be readily compared with the literature and hence aid identification [78].

Carmint (a mixture of coriander, lemon balm and spearmint extracts) has been demonstrated to reduce IBS symptoms [66]. Our findings with the essential oils of coriander seed, lemon balm and peppermint suggested that carmint’s efficacy may be due to antibacterial activity, and the ability to reverse gastrointestinal dysbiosis or SIBO. To investigate this further, extracts were made of coriander, lemon balm and spearmint leaves using a variety of solvents, and tested for their antibacterial activity. In each case, extracts made with ethanol or methanol displayed the most potent antibacterial activity, and since carmint is assumed to contain ethanolic extracts of the herbs, this suggests that carmint is effective against IBS because it has antibacterial activity. Other properties may also contribute to carmint’s mechanism of action, however, since both peppermint and lemon balm essential oils and extracts have been shown to have antispasmodic activity [57,58], peppermint has also been shown to act on serotonin receptors [121] and lemon balm reduces stress [122] and could therefore modulate the gut-brain axis.

Peppermint and lemon balm extracts are also present in the mixed herbal extract, Iberogast®, or STW-5, which has also been shown to be effective against IBS in a clinical trial [63]. This suggests that antibacterial activity may also underlie Iberogast®’s mechanism of action, in addition to its documented antispasmodic, anti-inflammatory, antioxidant and prosecretory effects [55,62]. This hypothesis is further strengthened by the fact that Iberogast® has been shown in clinical trials to be effective in treating functional dyspepsia [64], which has been linked to infection with *Helicobacter pylori* and can be treated with antibiotics [123].

There has been one case study that suggested that enteric-coated peppermint oil tablets could reduce SIBO. Treatment of a patient with IBS and SIBO with enteric-coated peppermint oil tablets for 20 days resulted in a 32% reduction in hydrogen and methane excretion at the 60 minute time-point of a lactulose breath test (six days after the end of treatment), which correlated with an improvement in the IBS symptoms of pain, bloating, eructation and altered bowel habit [124]. The was ascribed to an antibacterial effect of the peppermint oil in the capsules reducing bacterial numbers in the small intestine, although it has been suggested that the treatment may instead have altered gastrointestinal transit time [125]. It is debatable whether enteric-coated peppermint oil tablets would be able to reverse SIBO in all patients, however, because they are designed to uncoat in the lower intestine in order to exert an antispasmodic effect on the colon, and thus may traverse much of the small intestine intact. The type of enteric-coated peppermint oil capsules that were used in the study was not identified, which is relevant because different types vary in their pharmacokinetics. Mintec® tablets uncoat earlier with a lag time of 0.5 h and a time to peak release of 2.8 h, and display an intense release of peppermint oil, whereas Colpermin® tablets uncoat later with a lag time of 1.07 h and a time to peak release of 5 h and have a more steady release profile [126,127]. Thus Mintec® tablets would be more likely to be able to address SIBO. It could be argued that peppermint water [61] would be better still, although it would be important not to have too high a dose, since it could cause heartburn due to relaxation of the oesophageal sphincter [128], and also tachycardia [129]. Whether the patient whose case study was reported [124], had achlorhydria, which might have caused the enteric-coated peppermint oil tablets to uncoat early [130], was not addressed.

Some of the other herbs whose essential oils have been shown to have antibacterial properties in this current study are also present in herbal medicines that have been proposed to be useful for treating IBS, or used to treat digestive disorders in the past. Mandarin is present in the Chinese herbal medicine Tong Xie Yao Fang (TXYF), and a modified version TXYFa, which has been shown by a systematic review to be potentially effective in the treatment of IBS, since it reduced abdominal pain, distension, flatulence and diarrhoea for up to 6 months after treatment [131]. Absinthe contains fennel (shown in this study and others to have antibacterial properties) as well as wormwood (*Artemisia absinthium* L.), anise and often lemon balm, Roman wormwood (*Artemisia pontica* L.) and hyssop (*Hyssopus officinalis* L.) [132]. Although absinthe is now thought of as an alcoholic beverage, with unhappy connotations due to its ability to cause absinthism when drunk to excess, it was originally used in the 1780s to cure colic (aka IBS) and fight dysentery, which would correlate with its probable antibacterial activity.

## Conclusions

New treatments are needed for IBS. Since IBS has been linked to intestinal dysbiosis or small intestinal bacterial overgrowth, we have investigated the antibacterial activity of the essential oils of a range of culinary and medicinal herbs, many of which have traditionally been used as digestives or are present in herbal medicines that have been shown to be effective in treating IBS in clinical trials, in three assays: the disc diffusion assay, the turbidometric assay and the zone of clearance assay. Of the essential oils that are on the FDA list of essential oils that are generally recognised as safe to ingest, coriander seed, lemon balm and peppermint essential oils exhibited strong antibacterial activity in all three assays, suggesting that the reason why carmint is effective for treating IBS is because it has antibacterial activity. The fact that ethanolic extracts of coriander, lemon balm and spearmint leaves all exhibited antibacterial activity, provided more evidence to support this hypothesis. Since other herbal medicines that have been shown/proposed to be useful in treating IBS and other digestive disorders, contain essential oils or extracts with antibacterial activity, we hypothesise that antibacterial properties are an important mechanism of action for herbal IBS medicines. We consider that essential oils of coriander seed, lemon balm and peppermint and other antibacterial essential oils on the FDA GRAS list, as well as carmint and Iberogast® all warrant further investigation in mechanistic studies and randomised, double-blind, placebo controlled clinical trials to assess their ability to act on intestinal dysbiosis or SIBO and resolve the symptoms of IBS.

## Abbreviations

ANOVA: Analysis of variance; cfu: Colony forming units; DMSO: Dimethyl sulphoxide; FODMAPs: Fermentable oligosaccharides, disaccharides, monosaccharides and polyols; GC/MS: Gas chromatography with mass spectrometry; HPLC: High Purification Liquid Chromatography; IBS: Irritable bowel syndrome; LHBT: Lactulose hydrogen breath test; NICE: National Institute for Health and Clinical Excellence; NIST: The National Institute of Standards and Technology; PBM: Probability based matching; SIBO: Small intestinal bacterial overgrowth; VOC: Volatile organic compound.

## Competing interests

We have no competing interests to declare. Aiysha Thompson, Dilruba Meah, Nadia Ahmed, Rebecca-Conniff-Jenkins and Emma Chileshe were all undergraduate students at Swansea University while this work was carried out. Dr. Chris Phillips, Professor Tim Claypole, Dr. Dan Forman and Dr. Paula Row are all employed by Swansea University and received no funding for this work. Paula Row has since been the recipient of funding for summer vacation studentships from the British Society for Antimicrobial Chemotherapy and The Biochemical Society, respectively, for two other students to continue this work, and (together with Tim Claypole, Chris Phillips and Dr. Nidhika Berry, the director of the Public Health Laboratories in Singleton Hospital, Swansea) has also been awarded a “Bridging the Gaps” Grant from Funds awarded by EPSRC to Swansea University, to continue this work.

## Authors’ contributions

AT, DM and NA are all joint first authors. AT helped to design and carried out the turbidometric assays, designed and carried out the experiments with peppermint extracts, performed corroborative disc diffusion assays and zone of clearance assays, performed some of the statistical analysis, helped to prepare the figures, helped to write the manuscript, and critically reviewed the manuscript. DM helped to design and carried out the disc diffusion assays, designed the zone of clearance assays, performed the experiments with coriander and lemon balm extracts, performed corroborative turbidometric assays and zone of clearance assays, performed some of the statistical analysis, helped to write the manuscript, and critically reviewed the manuscript. NA carried out the zone of clearance assays, performed corroborative disc diffusion assays and turbidometric assays, performed some of the statistical analysis, helped to write the manuscript, and critically reviewed the manuscript. RC-J performed corroborative disc diffusion assays and turbidometric assays, helped to write the manuscript, and critically reviewed the manuscript. EC performed corroborative disc diffusion assays and turbidometric assays, helped to write the manuscript, and critically reviewed the manuscript. COP and TCC performed the GC/MS analysis, analysed the GC/MS data and helped to write the manuscript, and critically reviewed the manuscript. DWF helped carry out statisctical analysis of the data and provided critical intellectual input into the study and the manuscript, helped to write the manuscript and critically reviewed it. PER conceived and designed the study, helped to design the experiments, supervised AT, DM, NA, RCC and EC in carrying out the experiments, helped carry out statistical analysis of the data, helped to prepare the figures, took the lead in writing the manuscript, amalgamating the other authors’ contributions into it, and critically reviewed the manuscript. All authors read and approved the final manuscript.

## Pre-publication history

The pre-publication history for this paper can be accessed here:

http://www.biomedcentral.com/1472-6882/13/338/prepub
